# Starch intake and caries increment: A longitudinal study in Finnish adults

**DOI:** 10.1017/S1368980024002398

**Published:** 2024-11-26

**Authors:** Fariah H Jangda, Annaliisa L Suominen, Annamari Lundqvist, Satu Männistö, Ali Golkari, Eduardo Bernabé

**Affiliations:** 1 Institute of Dentistry, Queen Mary University of London, London, UK; 2 Institute of Dentistry, University of Eastern Finland, Kuopio, Finland; 3 Oral Health Teaching Unit, Kuopio University Hospital, Kuopio, Finland; 4 National Institute for Health and Welfare, Helsinki, Finland

**Keywords:** Starch, Carbohydrates, Dental caries, Longitudinal study, Adult, Finland

## Abstract

**Objective:**

To evaluate whether changes in starch intake (in terms of amount and food sources) were associated with increments in dental caries among adults.

**Design:**

This is an 11-year longitudinal study (2000–2011) with duplicate assessments for all variables. A 128-item FFQ was used to estimate intake of starch (g/d) and six starch-rich food groups (potatoes, potato products, roots and tubers, pasta, wholegrains and legumes). Dental caries was assessed through clinical examinations and summarised using the number of decayed, missing and filled teeth (DMFT score). The relationship between quintiles of starch intake and DMFT score was tested in linear hybrid models adjusting for confounders.

**Setting:**

Northern and Southern regions of Finland.

**Participants:**

922 adults, aged 30–88 years.

**Results:**

Mean starch intake was 127·6 (sd: 47·8) g/d at baseline and 120·7 (55·8) g/d at follow-up. Mean DMFT score was 21·7 (6·4) and 22·4 (6·2) at baseline and follow-up. Starch intake was inversely associated with DMFT score cross-sectionally (rate ratio for highest *v*. lowest quintile of intake: –2·73, 95 % CI –4·64, –0·82) but not longitudinally (0·32, 95 % CI –0·12, 0·76). By food sources, the intakes of pasta (–2·77, 95 % CI –4·21, –1·32) and wholegrains (–1·91, 95 % CI –3·38, –0·45) were negatively associated with DMFT score cross-sectionally but not longitudinally (0·03, 95 % CI –0·33, 0·39 and –0·10, 95 % CI –0·44, 0·24, respectively).

**Conclusion:**

Changes in the amount and sources of starch intake were not associated with changes in dental caries. Further studies should be conducted in different settings and age groups while focusing on starch digestibility and specific sources of starch.

Frequent consumption of free sugars increases the risk of dental caries by providing a substrate for acid-producing bacteria in the mouth^(1,2)^. Other types of carbohydrates, such as starch, are not fermentable by oral bacteria and pose no direct cariogenic risk. However, they can be broken down into sugars by the action of salivary amylase as soon as they are ingested, which raises the question of whether their intake could also be associated with dental caries^(3–5)^.

Starch is the predominant carbohydrate in the human diet and originates from plant storage organs, including seeds, fruits, roots and tubers^(6,7)^. Starch comes in multiple forms depending on the method of storage, cooking, processing, mixing with other foods and serving temperature, which alters its structure and properties^(6,8)^. This heterogeneity affects its digestion properties and absorption rates in the body^(8,9)^. Starch can be classified based on how rapidly it can be hydrolysed by enzymes. Starch-containing foods that are hydrolysed within 20 min to release glucose are categorised as rapidly digestible starch (RDS) and those that take over 20 min are categorised as slowly digestible starch (SDS). Any starch that remains undigested after 120 min is classified as resistant starch (RS)^(10,11)^. RDS is abundant in freshly cooked foods like bread and potatoes^(12)^. SDS is found in partially milled seeds and grains, as well as in dense foods such as pasta^(13)^. The primary sources of RS are wholegrains and legumes^(9,14)^. Focusing on RDS offers a more targeted approach to understanding the cariogenic potential of starch compared with solely examining total starch intake.

A systematic review found low-certainty evidence (three longitudinal studies involving children and one involving adults) indicating no association between total starch intake and dental caries^(15)^. The longitudinal study among adults showed that starch intake (quartiles) was not associated with root caries incidence among US men aged 47–90 years^(16)^. The review also found low-certainty evidence (two longitudinal studies involving children) suggesting a potential positive association between RDS intake and dental caries^(15)^. One study focused on processed starches consumed as snacks^(17)^, while the other examined sugar–starch interactions in foods^(18)^. Overall, given the limited evidence available and the heterogeneity between studies, the results from the primary studies were unsuitable for meta-analysis^(15)^. Another systematic review determined that regular between-meal consumption of processed foods containing both sugar and starch was associated with dental caries, based on findings from three cohort studies involving children^(5)^. However, the independent effect of starch on caries development could not be separated from that of sugars.

Oral conditions have remained relatively common in Finland in the new century^(19)^. Edentulousness decreased between 2000 and 2011 but remained high (11 %) in older adults (≥65 years). The prevalence of dental caries and deepened periodontal pockets (≥4 mm) has remained stable over time, with 22 % and 63 % of dentate adults being affected, respectively. There have been some changes in the use of dental services during the same period following the 2001–2002 reform. Since then, the whole Finnish adult population has been entitled to public dental services (PDS) with subsidised fees or private services have been partly reimbursed by the National Sickness Insurance (NSI). The use of both public and private services increased among older adults, whereas the use of public services increased especially among adults who came entitled to PDS services (aged 44 years or older)^(20)^. Furthermore, carbohydrate intake among 25–70-year-old adults has decreased slightly, with approximately one in five adults reporting <10 % change in intake over an average of 7 years^(21)^. There is also evidence that sugars intake is associated with caries increment among Finish adults^(22,23)^.

Most previous studies on this research area were carried out in children. The present study provides evidence from an adult population. Furthermore, it evaluates changes in both total starch intake and common sources of starch in the Finnish diet. The purpose of this 11-year longitudinal study was to evaluate whether changes in starch intake (in terms of amount and food sources) were associated with increments in dental caries among adults. It was hypothesised that the intake of total starch, and especially RDS, would be associated with caries increment.

## Materials and methods

### Study population

**Figure 1.**
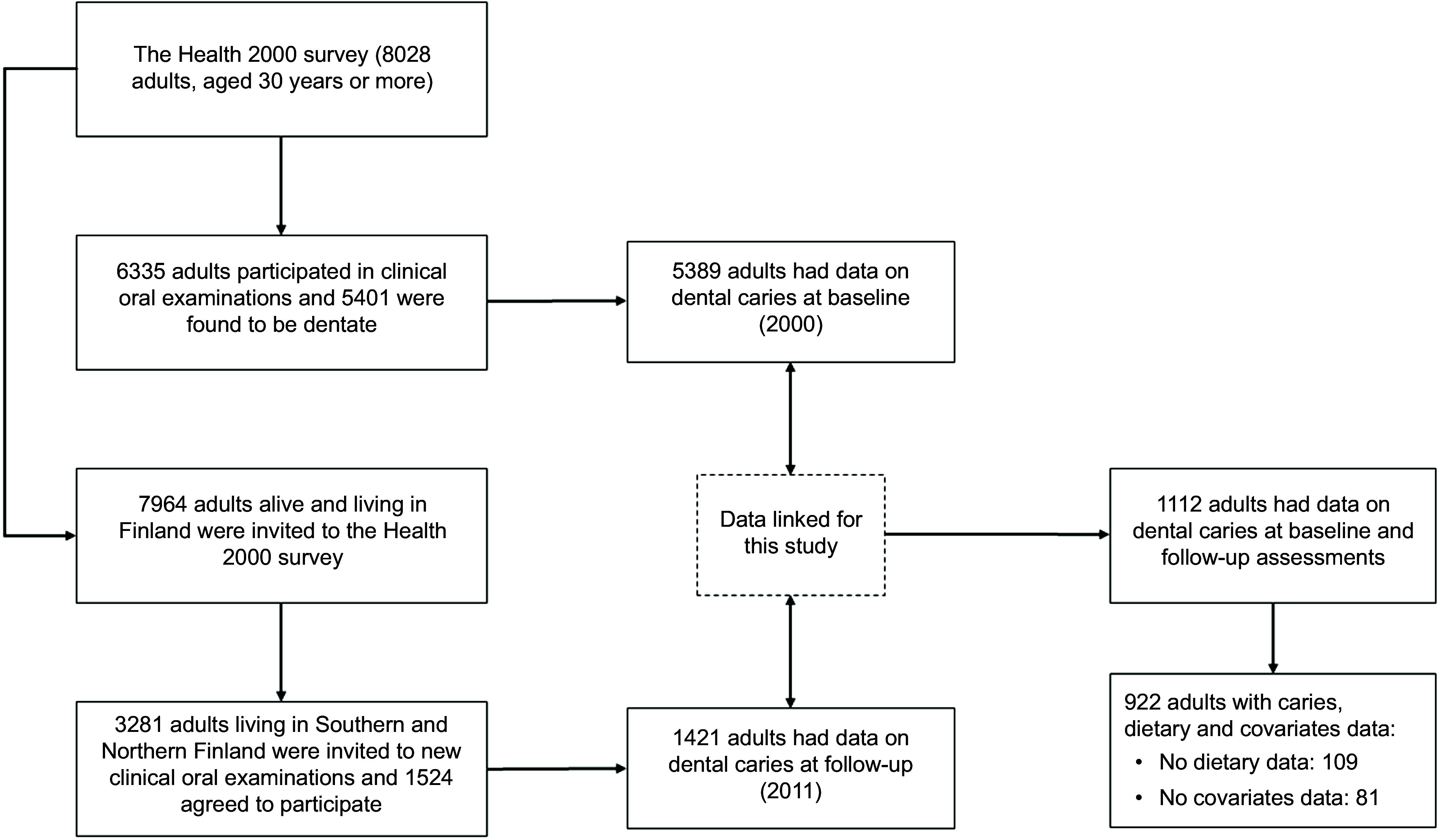
Selection of the study sample from adults participating in the Health 2000 and Health 2011 surveys.

This 11-year longitudinal study pooled data from individuals who took part in two national surveys carried out by the Finnish Institute for Welfare and Health (THL). Baseline data were from the main sample of the Health 2000 survey, which recruited 8028 adults aged 30 years or older living in mainland Finland. Overall, 6335 received dental examinations and 5401 were found to be dentate^(24)^. All participants of the Health 2000 survey alive and living in mainland Finland were invited to the Health 2011 survey, in which dental examinations were carried out in two of the five recruitment areas (Southern and Northern Finland), with 3281 adults invited to participate and 1524 re-examined (46 %)^(25)^. Adults aged 30 years or over at baseline, who participated in the two dental examinations, completed both FFQ and had complete information on all covariates, were included in the analysis (Fig. 1). No other selection criteria were used.

Ethical approval was sought for each survey separately, from the Ethics Committee at the Hospital District of Helsinki and Uusimaa. All participants provided written informed consent.

### Outcome data

Dental caries experience was the study outcome, which was determined from clinical dental examinations by trained dentists. Clinical examinations were carried out following the same protocol in the two surveys. Participants were seated on a dental chair and clinically examined using a fibre optic light, mouth mirror and WHO periodontal probe. Teeth were dried using compressed air and isolated with cotton rolls to facilitate visual inspection. A tooth was considered decayed if there was evidence of a carious lesion extending into dentin on any coronal or root surface, and the lesion was cavitated, penetrating the fissure and undermining the enamel, or the dentine walls were clearly softened^(26)^. The inter- and intra-examiner reliability for caries detection at tooth level was high, with Kappa values of 0·87 and 0·95, respectively, in the baseline survey^(27)^. Dental caries was summarised using the number of decayed, missing and filled teeth (DMFT score) and treated as a repeated measure in analysis. The DMFT score is a cumulative measure that captures past (fillings and extractions) and present (untreated disease) experience of dental caries. The DMFT score in an individual can vary from 0 (no caries experience) to 32 teeth (all teeth affected by caries).

### Exposure data

The exposure was starch intake, which was measured at baseline and follow-up. A validated semi-structured FFQ was used to measure habitual diet in the past year^(28–30)^. The FFQ comprised of 128 commonly used or nutritionally important food items and mixed dishes that were presented to participants in twelve sections: dairy products; cereals; spreads; vegetables; potatoes, rice, and pasta; meat; fish; chicken, turkey, and eggs; fruits and berries; desserts; sweets and snacks; and beverages. A standard portion size was assigned to each FFQ item using natural units (piece, slice, glass, tablespoon, etc.). Each FFQ item had nine response options to capture the frequency of food use (never or rarely, 1–3 times per month, once per week, 2–4 times per week, 5–6 times per week, once a day, 2–3 times a day, 4–5 times a day and more than 6 times a day). The FFQ was completed at home and sent to THL by post. When returned, they were checked for unreliable and missing answers by a nutritionist. Responses were used to derive the intakes of macronutrients (total starch intake and energy intake (EI)) and food groups using the Finnish Food Composition Database (Fineli®, THL, Finland)^(31)^. Total starch intake was analysed in quintiles of amount (g/d) and percent of energy intake (%EI). Based on existing literature^(7,12,32)^, six common food sources of starch were included in the analysis: (i) potatoes, (ii) potato products (i.e. French fries, potato chips), (iii) roots and tubers, (iv) pasta, (v) legumes and (vi) wholegrains (e.g. barley, oat and rye)^(33)^. All food sources were analysed as quintiles of amount consumed (g/d). Nutrient intakes were energy-adjusted using the residual method^(34)^.

### Covariate data

Information on sociodemographic characteristics (sex, age, education and marital status), health behaviours (physical activity, alcohol consumption, toothbrushing, dental flossing and dental attendance) and health conditions (BMI, self-rated health and history of chronic conditions) were collected in each survey and included as covariates in the analysis. Marital status was categorised as cohabiting (married and living with partner) or living alone (single, divorced or living apart and widowed). Physical activity was assessed with two questions on exercise during leisure time (LTE) for at least 30 min (so they felt at least slightly out of breath and sweating) and walking or cycling to work (WCW). Physical activity was categorised as ideal (LTE ≥ 4 times/week and WCW ≥ 30 min/d), sufficient (when only the LTE or WCW threshold was met), low (LTE = 2–3 times/week and WCW < 30 min/d) and sedentary (LTE ≤ 1 time/week and WCW < 30 min/d). Weekly alcohol intake (100 % ethanol) was categorised as no use, moderate use (women < 70 g, men < 140 g) and risk use (women ≥ 70 g, men ≥ 140 g)^(35)^. Toothbrushing was reported using a five-point ordinal scale and categorised as twice or more daily, once daily, or less than daily. Dental flossing was reported using a four-point ordinal scale and categorised as daily, less than daily or never. Regular/habitual dental attendance was reported using three response options and categorised as for check-ups or only when in trouble (including those who had never visited the dentist). Participants’ weight and height were measured by trained nurses using a wall-mounted stadiometer and a bioimpedance device’s scale (InBody 3.0, Biospace), respectively. Participants were classified as normal weight (BMI < 25 kg/m^2^), overweight (25–29·9) or obese (≥30). Current general health status was reported on a five-point ordinal scale and categorised as poor, moderate or good. Participants also reported if they had ever been diagnosed by a physician with diabetes, heart disease, hypertension or stroke.

### Statistical analysis

All analyses were performed in Stata MP 18 (StataCorp LP). First, the DMFT score at baseline and follow-up were compared by covariates. Then, the DMFT score at baseline and follow-up were compared by quintiles of total starch intake. Student’s *t* test and the test for linear trends were used to compare the DMFT score between unordered and ordered groups, respectively.

The relationship between total starch intake and DMFT score was analysed using a linear hybrid model that accounted for both time-invariant (sex) and time-variant covariates (education, marital status, physical activity, alcohol consumption, BMI groups, history of hypertension, diabetes, heart disease and stroke, self-rated health, toothbrushing frequency, dental flossing, dental attendance and EI). Age was disaggregated into two components, baseline age (10-year brackets) and a binary indicator for time (coded as 0 for baseline and 1 for follow-up). Hybrid models combine the advantages of fixed-effects models (i.e. estimating within-individual changes and controlling for unmeasured time-invariant factors) and random-effects models (i.e. controlling for time-invariant predictors), making them suitable for handling correlated panel data with an unbalanced structure and missing observations^(36,37)^. The impact of time-variant predictors is separated into two components: (i) between-person regression coefficients representing the average value across all assessments within individuals and (ii) within-person regression coefficients representing variations around the individual-specific mean^(36,38)^. Between-person estimates reflect differences in the DMFT score between quintiles of starch intake at any given time point (i.e. cross-sectional associations), while the within-person estimates reflect the changes in DMFT score attributed to changes in quintiles of starch intake between the two time points (i.e. longitudinal associations). A random intercept for DMFT score was incorporated to address the correlated nature of the data (repeated assessments). The same model was used for each of the six sources of starch evaluated. All models were run in Stata using the xthybrid suite^(39)^. The Hausman’s test was used to evaluate the equivalence of between- and within-person estimates^(37)^. If the null hypothesis is rejected, within-person estimates are different from between-person estimates^(39)^.

In sensitivity analysis, all models were repeated using the number of decayed and filled teeth (DFT) score instead of the DMFT score, which excludes the missing teeth from the calculation. The DFT is used to minimise the impact of measurement error when adjudicating whether a tooth was missing due to caries or other reasons (congenital, trauma, orthodontic treatment, periodontitis, etc.). It can also vary from 0 to 28 teeth (excluding third molars).

## Results

There were 1112 dentate adults in the Health 2000 survey who were clinically examined in the Health 2011 Survey. Of them, 190 were excluded because of missing data on starch intake (*n* 109) and covariates (*n* 81). Therefore, the final sample included 922 adults with complete data on all relevant variables at baseline and follow-up (Fig. 1). Retained participants were older and more educated and have more favourable behaviours and better self-rated health than those lost to follow-up. The mean DMFT score was 21·69 ± 6·39 and 22·43 ± 6·15 teeth at baseline and follow-up, respectively. Older adults, those with lower education, no alcohol consumption and poorer self-rated health and those with history of hypertension, diabetes and heart disease had greater DMFT scores than their respective counterparts at both baseline and follow-up (Table 1). Participants with greater BMI and those with history of stroke had greater DMFT scores than those of normal weight at baseline, whereas participants living alone had greater DMFT scores than those cohabiting at follow-up. The mean total starch intake was 127·61 ± 47·76 g/d at baseline and 120·71 ± 55·78 at follow-up (Table 2). Potatoes, wholegrains and roots and tubers were the main sources of starch in both surveys.

**Table 1.** Dental caries levels at baseline and follow-up according to covariates. The Health 2000 and Health 2011 surveys of adults 30 years or over in Finland (*n* 922)

Covariates	DMFT at baseline (*n* 922)				DMFT at follow-up (*n* 922)			
	*n*	Mean	sd	*P* value^*^	*n*	Mean	sd	*P* value^*^
*Sex*				0·575				0·762
Male	400	21·6	6·4		400	22·3	6·1	
Female	522	21·8	6·4		522	22·5	6·2	
*Age at baseline*				<0·001				<0·001
30–39 years	298	16·8	6·2		298	17·9	6·0	
40–49 years	269	23·0	5·1		269	23·6	5·0	
50–59 years	244	24·4	4·8		244	24·9	4·7	
60–69 years	96	25·3	4·8		96	25·9	4·8	
70+ years	15	26·8	5·9		15	28·3	4·0	
*Education*				<0·001				<0·001
Basic	169	24·5	5·3		161	25·2	5·2	
Secondary	314	22·1	6·3		284	23·0	6·1	
Higher	439	20·3	6·5		477	21·2	6·1	
*Marital status*				0·208				0·012
Cohabiting	717	21·5	6·3		699	22·1	6·2	
Living alone	205	22·2	6·7		223	23·3	5·9	
*Physical activity*				0·103				0·494
Sedentary	316	21·2	6·3		262	22·7	6·2	
Low	305	21·8	6·8		347	22·3	6·0	
Sufficient	257	22·1	6·2		281	22·3	6·3	
Ideal	44	22·1	5·7		32	22·2	6·5	
*Alcohol consumption*				0·009				0·004
No use	84	24·7	5·4		120	24·2	5·9	
Moderate use	656	21·3	6·5		617	22·2	6·3	
Risk use	182	21·6	6·2		185	21·9	5·7	
*BMI group*				<0·001				0·091
Normal	415	20·9	6·6		346	22·4	6·3	
Overweight	359	21·8	6·4		363	21·7	6·2	
Obese	148	23·6	5·5		213	23·6	5·6	
*Diabetes*				0·045				0·009
No	905	21·6	6·4		865	22·3	6·1	
Yes	17	24·8	6·3		57	24·5	6·0	
*Heart disease*				<0·001				0·001
No	758	21·3	6·4		791	22·1	6·2	
Yes	164	23·6	6·2		131	24·1	5·7	
*Hypertension*				<0·001				<0·001
No	708	21·1	6·5		644	21·7	6·2	
Yes	214	23·8	5·6		278	24·1	5·6	
*Stroke*				0·042				0·068
No	910	21·6	6·4		899	22·4	6·2	
Yes	12	25·4	4·0		23	24·7	5·7	
*Self-rated general health*				<0·001				0·001
Poor	32	25·5	4·3		754	22·0	6·1	
Moderate	181	23·6	5·8		128	24·6	6·2	
Good	709	21·0	6·5		40	23·0	6·2	
*Toothbrushing*				0·914				0·634
Twice or more daily	663	21·7	6·3		699	22·4	6·1	
Once daily	231	21·5	6·5		200	22·6	6·2	
Less than daily	28	22·2	6·4		23	22·6	6·0	
*Dental flossing*				0·708				0·101
Daily	127	23·3	6·0		272	23·3	5·8	
Less than daily	427	20·6	6·3		405	21·8	6·0	
Never	368	22·4	6·4		245	22·5	6·6	
*Dental attendance*				0·469				0·455
For check-ups	611	21·8	6·1		645	22·5	5·9	
Only when in trouble	311	21·5	6·9		277	22·2	6·6	

DMFT, the number of decayed, missing and filled teeth.

* Student’s *t* test and test for linear trends were used to compare unordered and ordered categories, respectively.

**Table 2.** Measures of starch intake. The Health 2000 and Health 2011 surveys of adults 30 years or over in Finland (*n* 922)

	Starch (g/d)	Starch (%EI)	Potatoes (g/d)	Potato products (g/d)	Roots and tubers (g/d)	Pasta (g/d)	Legumes (g/d)	Wholegrains (g/d)
*Intake at baseline*								
Mean	127·61	23·23	150·27	5·31	49·89	7·48	13·84	60·42
sd	47·76	4·96	100·82	5·30	44·64	7·66	13·95	39·40
Median among quintiles								
Q1 (lowest)	72·63	17·21	57·27	0·79	13·46	2·97	2·81	12·37
Q2	99·40	20·83	100·15	3·33	24·60	3·02	6·11	30·56
Q3	120·97	23·07	131·14	4·47	39·97	6·34	9·88	57·15
Q4	147·31	25·36	173·77	5·72	61·15	6·67	14·52	79·53
Q5 (highest)	190·86	29·63	228·62	9·39	90·19	19·07	34·19	121·99
*Intake at follow-up*								
Mean	120·71	21·35	113·33	5·89	45·51	5·61	19·34	73·27
sd	55·78	5·74	85·38	8·77	47·11	5·95	21·18	46·66
Median among quintiles								
Q1 (lowest)	59·07	14·64	35·92	0·10	11·03	0·10	4·84	20·01
Q2	90·08	18·56	66·04	0·63	19·95	2·18	9·29	41·26
Q3	113·76	21·43	95·19	4·23	35·33	4·46	13·58	70·02
Q4	141·68	24·03	131·27	7·05	50·33	5·83	20·64	90·53
Q5 (highest)	189·31	28·26	197·33	11·83	84·95	13·48	37·59	134·92

Q, Quintiles; %EI, percent of energy intake.

Dietary data were collected using a FFQ. Intakes (g/d) are reported without adjustment for energy intake.

No cross-sectional association was found between starch intake and the DMFT score at baseline or follow-up (Table 3). This was irrespective of whether starch intake was expressed in g/d or as %EI. Results from the hybrid models are presented in Table 4. Total starch intake (g/d) was inversely associated with the DMFT score, although this association was mainly driven by between-person (cross-sectional) differences. Cross-sectional estimates showed that adults in the third, fourth and fifth (highest) quintile of intake had a mean DMFT score lower by –1·56 (95 % CI –3·02, –0·10), –2·01 (95 % CI –3·53, –0·49) and –2·73 (95 % CI –4·64, –0·82) teeth than those in the first (lowest) quintile. The longitudinal estimates (within-person differences) showed that changes in starch intake were not associated with increments in the DMFT score. Similar results were observed with starch intake as %EI. Cross-sectional estimates showed that adults in the third, fourth and fifth (highest) quintile of intake had a mean DMFT score lower by –1·63 (95 % CI –3·05, –0·20), –1·07 (95 % CI –2·50, 0·35) and –2·24 (95 % CI –3·62, –0·86) teeth than those in the first (lowest) quintile. Longitudinal estimates showed that changes in starch intake were not associated with increments in DMFT score.

**Table 3.** Crude cross-sectional associations between total starch intake (g/d) and DMFT score at baseline and follow-up. The Health 2000 and Health 2011 surveys of adults 30 years or over in Finland (*n* 922)

Starch intake	DMFT at baseline			DMFT at follow-up		
	*n*	Mean	sd	*n*	Mean	sd
*Starch (g/d)*						
Q1 (lowest)	185	21·16	6·36	185	22·62	5·73
Q1	184	22·34	6·59	184	22·29	6·57
Q3	185	21·09	6·46	185	22·64	6·15
Q4	184	21·70	6·16	184	22·03	6·24
Q5 (highest)	184	22·16	6·33	184	22·54	6·08
P value for trend^*^		*0·356*			*0·765*	
*Starch (%EI)*						
Q1 (lowest)	185	21·78	5·94	185	22·90	5·91
Q1	184	20·98	6·72	184	21·92	5·65
Q3	185	21·05	6·54	185	22·28	6·91
Q4	184	22·05	6·48	184	22·52	6·01
Q5 (highest)	184	22·60	6·17	184	22·50	6·23
P value for trend^*^		*0·069*			*0·882*	

DMFT, the number of decayed, missing and filled teeth; Q, quintiles; %EI, percent of energy intake.

* Tests for linear trend were derived from linear regression models.

**Table 4.** Linear hybrid models for the association between starch (amount and main sources) and DMFT score. The Health 2000 and Health 2011 surveys of adults 30 years or over in Finland (*n* 922)

	Between-person effects*		Within-person effects*		Hausman’s test †
	Coef.	95 % CI	Coef.	95 % CI	
*Starch (g/d)*					
Q1 (lowest)		Reference		Reference	
Q2	–0·19	–1·61, 1·23	0·16	–0·12, 0·45	0·633
Q3	–1·56	–3·02, –0·10	0·24	–0·07, 0·55	0·018
Q4	–2·01	–3·53, –0·49	0·07	–0·29, 0·43	0·009
Q5 (highest)	–2·73	–4·64, –0·82	0·32	–0·12, 0·76	0·002
*Starch (%EI)*					
Q1 (lowest)		Reference		Reference	
Q2	–1·31	–2·81, 0·18	0·00	–0·26, 0·26	0·090
Q3	–1·63	–3·05, –0·20	0·24	–0·04, 0·51	0·012
Q4	–1·07	–2·50, 0·35	0·07	–0·21, 0·35	0·123
Q5 (highest)	–2·24	–3·62, –0·86	0·08	–0·23, 0·40	0·001
*Potatoes (g/d)*					
Q1 (lowest)		Reference		Reference	
Q2	0·54	–0·87, 1·94	0·29	0·01, 0·58	0·74
Q3	0·87	–0·55, 2·30	0·33	0·03, 0·63	0·464
Q4	1·10	–0·32, 2·51	0·45	0·13, 0·76	0·381
Q5 (highest)	0·14	–1·37, 1·65	0·71	0·36, 1·06	0·471
*Potato products (g/d)*					
Q1 (lowest)		Reference		Reference	
Q2	0·14	–1·32, 1·59	0·07	–0·19, 0·34	0·931
Q3	–0·64	–2·16, 0·87	–0·07	–0·34, 0·20	0·465
Q4	–0·17	–1·73, 1·39	–0·02	–0·29, 0·25	0·849
Q5 (highest)	–1·05	–2·78, 0·68	0·11	–0·18, 0·39	0·195
*Roots and tubers (g/d)*					
Q1 (lowest)		Reference		Reference	
Q2	–0·15	–1·57, 1·26	–0·18	–0·47, 0·10	0·966
Q3	0·04	–1·38, 1·47	0·12	–0·18, 0·41	0·921
Q4	–1·01	–2·43, 0·40	0·04	–0·27, 0·35	0·154
Q5 (highest)	–0·23	–1·67, 1·21	0·19	–0·15, 0·54	0·572
*Pasta (g/d)*					
Q1 (lowest)		Reference		Reference	
Q2	–0·33	–1·71, –1·05	–0·03	–0·31, 0·25	0·677
Q3	–1·12	–2·37, 0·13	–0·13	–0·40, 0·13	0·131
Q4	–1·80	–3·33, –0·27	0·04	–0·29, 0·36	0·022
Q5 (highest)	–2·77	–4·21, –1·32	0·03	–0·33, 0·39	<0·001
*Legumes (g/d)*					
Q1 (lowest)		Reference		Reference	
Q2	1·10	–0·34, 2·53	–0·18	–0·45, 0·09	0·088
Q3	1·21	–0·18, 2·60	–0·22	–0·50, 0·06	0·048
Q4	–0·50	–1·92, 0·91	–0·08	–0·38, 0·22	0·565
Q5 (highest)	–0·01	–1·47, 1·46	–0·03	–0·34, 0·29	0·978
*Wholegrains (g/d)*					
Q1 (lowest)		Reference		Reference	
Q2	0·46	–0·94, 1·87	0·00	–0·29, 0·28	0·523
Q3	–0·39	–1·77, 0·98	0·03	–0·27, 0·33	0·556
Q4	–0·64	–2·06, 0·78	0·01	–0·31, 0·31	0·385
Q5 (highest)	–1·91	–3·38, –0·45	–0·10	–0·44, 0·24	0·018

DMFT, the number of decayed, missing and filled teeth; Q, quintiles.

* A linear hybrid model was fitted for the DMFT score and regression coefficients reported. Models were adjusted for sex, age groups, marital status, education, alcohol intake, physical activity, BMI group, history of hypertension, diabetes, heart disease and stroke, self-rated health, toothbrushing, dental flossing, dental attendance and the time indicator (coded as 0 for baseline and 1 for follow-up).

† The Hausman’s test was used to evaluate the equivalence of between- and within-person estimates.

By food groups, the intake of pasta and wholegrains were negatively associated with the DMFT score, although these relationships were driven by between-person differences (Table 4). For pasta, adults in the highest and second highest quintile of intake had a mean DMFT score lower by –1·80 (95 % CI –3·33, –0·27) and –2·77 (95 % CI –4·21, –1·32) teeth than those in the lowest quintile of intake. For wholegrains, adults in the highest quintile of intake had a mean DMFT score lower by –1·91 (95 % CI –3·38, –0·45) teeth than those in the lowest quintile of intake. Notably, changes in the intake of pasta and wholegrains (within-person estimates) were not associated with increments in the DMFT score.

Similar findings were observed in sensitivity analysis using the DFT score instead of the DMFT score. Total starch intake and the intake of pasta and wholegrains were inversely associated with the DFT score cross-sectionally but not longitudinally.

## Discussion

This study included Finnish dentate adults within a large age range (i.e. 30–88 years). The caries increment among participants was relatively low, with an average of less than one tooth per adult developing new disease over 11 years. This contrasts with the high caries experience observed at baseline (mean DMFT score: 21·7 out of 32 teeth affected by the disease). These figures are consistent with those reported in the adult population of Finland and other European countries^(19)^.

This study provides no support for the association between changes in starch intake and changes in dental caries among Finnish adults. A cross-sectional association between starch intake and dental caries was found after accounting for covariates, but the same association was not observed longitudinally. The fact that the cross-sectional association was not replicated longitudinally points to the role of unmeasured confounding (omitted variable bias) in between-person estimates^(37)^. Contrarily, within-person estimates use individuals as their own controls (i.e. comparing DMFT scores before and after they change their starch intake), which removes the effect of time-invariant confounders (both measured and unmeasured) while also controlling for time-varying confounders included in the model^(36,39)^. By emphasising within-person effects over between-person effects, this study was able to address confounding more efficiently and provided a more accurate assessment of how changes in starch intake affect caries risk. The current finding confirms those from the only previous longitudinal study (mean follow-up time: 11 ± 5 years) which found no association between starch intake (%EI) and incidence of root caries among 533 male US adults aged 47–90 years^(16)^. This is noteworthy given the higher energetic intake from starch in the US study than in ours (28 % *v*. 21–23 %). An alternative explanation for the cross-sectional but not longitudinal association between starch and caries is reverse causality. Dental caries is a lifelong progressive and cumulative disease. It is thus possible that adults with low caries experience have been on a healthy dietary trajectory containing less sugars and more starch and fibre over the life course.

A second finding of this study was related to common sources of starch intake and their association with dental caries. Common sources of RDS in the Finnish diet (potatoes, roots and tubers) were not associated with dental caries. This finding challenges the hypothesis of Halvorsrud, Lewney^(15)^ which posits that RDS could increase the risk of dental caries. As for SDS, the intakes of pasta and wholegrains were inversely associated with dental caries cross-sectionally but not longitudinally. Similar to the findings for total starch intake, differences in the between-person and within-person estimates suggest the role of unmeasured confounding. This interpretation is further supported by the lack of association between legumes intake, another source of SDS and dental caries.

There are no specific recommendations for how much starch people should consume. However, the WHO^(40)^ recommends that most carbohydrates come from wholegrains, vegetables, fruits and pulses. These recommendations have been endorsed by the European Food Safety Authority (EFSA)^(41)^ and the Nordic Council of Ministers^(42)^. In addition, the British Scientific Advisory Committee on Nutrition (SACN) recommended replacing simple (sugars) with complex carbohydrates^(43)^. Health professionals, including dentists, need to consider these recommendations when discussing lifestyle changes with patients as a healthy diet is an important factor to prevent multiple non-communicable diseases^(44,45)^. In terms of research, future prospective studies should evaluate other common sources of dietary starch, noting that these might vary across settings and age groups. Measuring starch digestibility, the quality of carbohydrate intake (as indicated by the glycaemic index and glycaemic load) and distinguishing between simple and complex carbohydrates could provide valuable insights on this research field. Based on the within-group difference in DMFT score observed among adults in the highest quintile of starch intake (coefficient: 0·32, 95 % CI –0·12, 0·76, sd for the difference: 6·82, CI width: 0·88), it is estimated that a sample of 1939 individuals would be required to be 90 % certain that the CI width in the future study will be no larger than a width of 0·62 (which will make the lower limit of the CI above zero), assuming a 5 % significance level.

The study suffers from some limitations. First, there was moderate attrition between the baseline and follow-up surveys. Retained participants were more likely to have more education, more favourable behaviours and better health. In addition to this, some participants were excluded because of missing data on relevant variables. The study also focused on adults, meaning that findings should not be extrapolated to other age groups. Taken together, this means that caution must be exercised when generalising the results. Second, dental caries was measured using the DMFT score, which although widely used in oral health surveys it is not without limitations. One important limitation is the accuracy in adjudicating whether teeth were missing due to dental caries or other reasons. However, similar findings were observed when using the DFT score instead of the DMFT score. Third, accurately assessing dietary intake poses challenges, with self-reported data from FFQ, albeit validated, susceptible to measurement error and underestimation. FFQ are commonly used in epidemiological surveys as they are more feasible to perform than food recalls but cannot be used to derive average intakes. To address this issue, participants were grouped according to quintiles of intake, which allowed making comparison between different levels of habitual intake of starch. Future studies should address these limitations and refine methods to determine starch digestibility for deeper insights into the relationship between starch intake and dental caries.

In conclusion, this 11-year longitudinal study among Finnish adults found no association between changes in the amount and sources of starch intake and changes in dental caries. The present findings challenge the hypothesised role of starch in caries development. Future research should evaluate the relationship between starch intake and dental caries in different settings and age groups while also focusing on starch digestibility and specific sources of starch in the diet.
